# Posterior reversible encephalopathy in a pregnant woman without preeclampsia or eclampsia: A case report

**DOI:** 10.1097/MD.0000000000030519

**Published:** 2022-09-09

**Authors:** Yi Zhang, Bing Liang, Cuiping Zhao, Yi Zhou, Chuanzhu Yan

**Affiliations:** a Department of Neurology, Rizhao Central Hospital, Shandong, China; b Department of Neurology, Qilu Hospital (Qingdao), Cheeloo College of Medicine, Shandong University, Qingdao, China; c Department of Ultrasonic, Rizhao Hospital of Traditional Chinese Medicine, Rizhao, China.

**Keywords:** encephalopathy, posterior reversible encephalopathy syndrome, pregnancy

## Abstract

We report a case of a pregnant woman who presented posterior reversible encephalopathy syndrome (PRES) without pre-eclampsia, eclampsia, or any other common causes of PRES.

**Methods:**

A 32-year-old primigravida at 25 weeks and 4 days of gestation was admitted to neurology department because of suffering intermittent headache, hearing loss, memory loss with mental and behavioral disorder, and blurred vision for 1 month. She was healthy before without hypertension, migraine, or other medical or family history. Brain magnetic resonance imaging (MRI) revealed diffuse symmetrical high-signal intensity lesions in the white matter, medulla oblongata, without enhancement. After completely multidisciplinary discussion and with the family of the patient, she accepted termination of pregnancy.

**Results:**

After the operation, the patient improved symptomatically. The follow-up MRI showed a decrease of the white matter lesion after 3 months and complete recovery at postoperative 6 months. The patient returned to work without any neurological sequelae.

**Conclusion:**

It might widen the cause spectrum of PRES that pregnancy itself without pre-eclampsia, eclampsia, or any other known risk factors could cause PRES. Pregnancy with acute or subacute leukoencephalopathy should be screened related causes and risk factors carefully. Hormonal fluctuations during the pregnancy might account for pregnancy-related PRES.

## 1. Introduction

Posterior reversible encephalopathy syndrome (PRES) was first reported in 1996 by Judy Hinche.^[1]^ Pre-eclampsia, eclampsia, hypertension, immunosuppressive medicines after transplantation, cancer chemotherapy, and autoimmune diseases are the common causes of PRES.^[2]^ On neuroimaging, the most common lesions of PRES are located at parietal and occipital lobes in up to 50% of cases, the superior frontal sulcus up to 27%, both anterior and posterior, medial and lateral watershed zones up to 29%, the deep white matter, basal ganglia, thalami, brainstem, and pons up to 13%.^[3]^ Here, we report clinical and radiographic course of a patient who was diagnosed with PRES related to pregnancy, without eclampsia, pre-eclampsia, and other medical history.

## 2. Case presentation

Patient has provided informed consent for publication of the case. The study was performed in accordance with the code of ethics of the world medical association (declaration of Helsinki) for experiments involving humans and the protocol was approved by the research ethics committee of Qilu Hospital of Shandong University.

**Figure 1:**
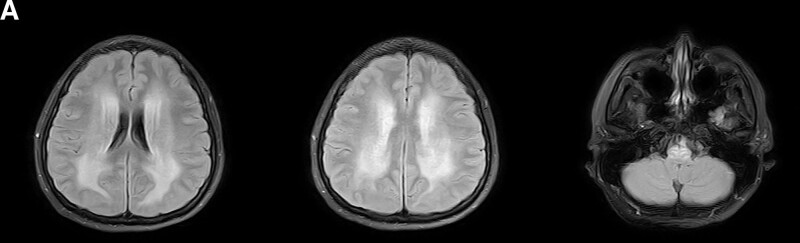
. T2 fluid-attenuated inversion recovery magnetic resonance imaging (MRI) on admission shows diffuse bilateral, symmetric hyperintense signal abnormality involving both white matter, medulla oblongata, and brainstem (A).

A 32-year-old primigravida at 25 weeks and 4 days of gestation was admitted to neurology department because of suffering intermittent headache, hearing loss, memory loss with mental and behavioral disorder, and blurred vision for 1 month. The patient’s condition became worse gradually and she became lethargy. She was healthy before without hypertension, migraine, or any other medical history or family history. On examination, her blood pressure was 120/84 mm Hg with 80 heartbeat/min and 98% of oxygen saturation. Her orientation was bad but she was alert, pupils were reactive to light. Other neurological examinations were normal. Laboratory tests including complete blood count, renal and liver function, electrolytes, blood sugar, clotting parameters, antinuclear antibody, rheumatic arthritis factor, complement 3 and 4 levels, and antiphospholipid antibody were normal. Cerebrospinal fluid analysis showed normal with 1 lymphocyte/ml, 0.38 g/L of protein but the pressure was 280 mmH_2_O. Brain magnetic resonance imaging (MRI) showed diffuse symmetrical high-signal intensity lesions on diffusion-weighted imaging, T2-weighted imaging, and T2 fluid-attenuated inversion recovery in the white matter, medulla oblongata, without enhancement (Fig. 1A). The loss of flow void in right transverse sinus and right sigmoid sinus in magnetic resonance venography (MRV) indicated the possible thrombosis.

**Figure 2:**
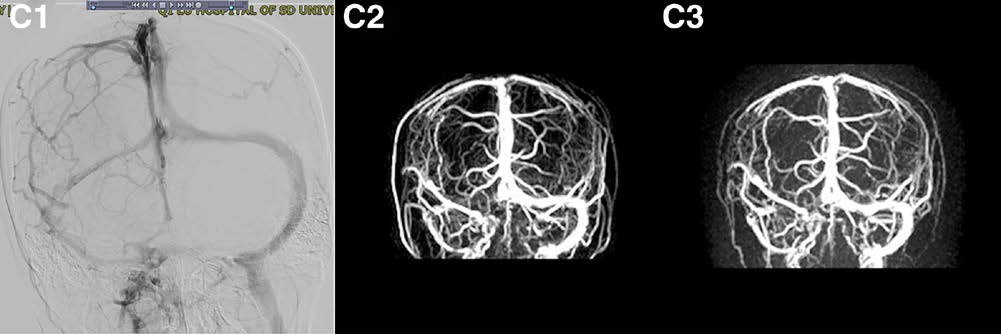
. MRV on admission indicated the possible thrombosis in right transverse sinuses and right sigmoid sinuses (C1). There still was stenosis of right transverse sinus in MRV after 3 months (C2) and 6 months postoperatively (C3). MRV= magnetic resonance venography.

**Figure 3:**
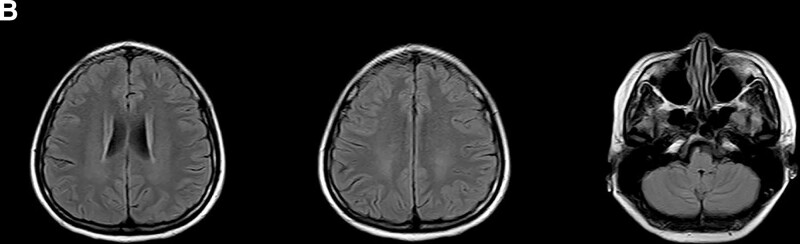
. The follow-up MRI showed a decrease of the white matter lesion after 3 months and complete recovery at postoperative 6 months (B). MRI = magnetic resonance imaging.

Because of high intracranial pressure and MRV manifestation, digital subtraction angiography (DSA) was performed and the result showed stenosis and mild thrombosis in the right transverse sinus (Fig. 2C1) with normal cerebral circulation time. Based on the MRI findings, screening for potential pathogenic medicines was performed and further laboratory test for toxic substance, tumor markers, blood and urine organic acids, and gene for hereditary cerebral leukodystrophy were ordered, but there were no positive findings. Treatment with mannitol (50 mg qid), dexamethasone (10 mg/d), and low molecular weight heparin (60 mg/d) was started on the day of admission to decrease the pressure of cerebrospinal fluid and treat the possible thrombosis of sinus. The patient’s condition became worse gradually and she became unconscious. After complete multidisciplinary discussion and with the family of the patient, she accepted termination of pregnancy. After the operation, she improved symptomatically and the intracranial pressure decreased to normal. The follow-up MRI showed a decrease of the white matter lesion after 3 months and completely recover at postoperative 6 months (Fig. 3B). There was slight stenosis of right transverse sinus in MRV (Fig. 2C2 and C3). She returned to work without any neurological sequelae. The diagnosis of the patient was consistent with PRES-related pregnancy without common causes.

## 3. Discussion

PRES should be considered when we have a pregnant woman who has sudden onset of headaches, seizures, vomiting, and altered mental status. Differential diagnoses should include vascular diseases such as cerebral venous sinus thrombosis (CVST), toxic or metabolic leukoencephalopathy, hereditary leukodystrophy, demyelinating disease of central nervous system, tumors such as lymphoma and gliomatosis, and reversible vasoconstriction syndromes.

CVST typically present with headache, vomiting, vision loss because of intracranial hypertension or variable focal neurological impairment due to heterogeneous draining vein. DSA provides definitive diagnosis.^[4]^ But in our case, the extensive diffused lesion could not be explained with CVST, because there was only mild thrombosis in right transverse sinus and the circulation time in DSA was normal. Furthermore, therapy with anticoagulation and anti-high intracranial pressure agent did not improve the symptoms of the patients. So CVST was not the real cause of such diffuse symmetrical high-signal intensity lesions of the patient.

We excluded other diseases, which should be differentiated through screening toxic substance, blood and urine organic acids, gene for hereditary cerebral leukodystrophy, and enhanced MRI.

To the best of our knowledge, there is only one case reported that pregnant patients with PRES without either of pre-eclampsia or eclampsia.^[5]^ We made the diagnosis of PRES related to pregnancy itself based on her clinical course, exclusion of other presumable diseases, radiological findings on MRI, and complete recovery of cerebral white matter lesion only after termination of pregnancy. Pregnancy alone without pre-eclampsia and eclampsia might be one cause of PRES.

Although the exact etiopathogenesis of PRES is not fully understood, a couple of theories of pathophysiological mechanisms have been postulated. The most popular theory is that rapidly developing hypertension exceeds the upper limit of cerebral blood flow autoregulation causes hyperperfusion, disruption of the blood–brain barrier, and subsequent vasogenic edema.^[6]^ However, in about 20% to 50% of patients with PRES cases, normotensive or hypotensive was found.^[7]^ Proposed theories such as modified immune mechanisms, placental abnormalities, oxidative stress, dysfunction of endothelial cells, dietary characteristics, and genetic susceptibility are thought to play a role in the pathogenesis of pregnancy-related PRES. We postulated that hormonal fluctuations of gonadal hormones levels, estrogens and progesterone, through modulating calcitonin gene-related peptide, serotonin and prostaglandins, some of which have been proved to be associated with vasoconstriction following menstrual migraine.^[7]^ might be involved in alterations in cerebral vascular tone. The alterations in cerebral vascular tone lead to endothelial injury with increased endothelial permeability and secondary vasogenic edema. On the other hand, the significant increase of ovarian steroid hormone levels throughout pregnancy leads to severe cerebral vasoconstriction with subsequent hypoperfusion and breakdown of the blood brain barrier that might cause cerebral edema.^[8]^ Multicenter studies with large sample sizes are still needed to clarify the pathogenesis of PRES secondary to pregnancy.

The prognosis of PRES is usually favourable, which is mainly determined by timely and adequate treatment of the underlying disease. Despite 70% to 90% of PRES patients can wholly recover in the range of 2 to 8 days,^[9]^ poor outcomes such as cerebral hemorrhage or ischemia and irreversible neurological deficits, death occurred in 26% to 36% of patients, and death was found in 8% to 17%.^[10]^ Occasionally, a few patients take several weeks to completely recover. Early diagnosis and adequate treatment of PRES are imperative to gain better prognosis. Furthermore, the outcome of the PRES is also dependent on the causative factors that women with PRES in pre-eclampsia and eclampsia have a better outcome than patients with other etiological factors. Studies have shown that about 5% to 10% of patients develop recurrent PRES, which is more frequent in patients with uncontrolled hypertension.^[11]^

There are three teaching points in this case. First, pregnancy without pre-eclampsia, eclampsia, or any other known risk factors could cause PRES, which might widen the cause spectrum of PRES. Second, pregnancy with acute or sub-acute diffuse leukoencephalopathy should be screened associated causes and risk factors carefully. The hormonal fluctuations during the time of pregnancy might account for pregnancy-related PRES. Our case will remind physicians that pregnancy-related PRES should be considered when there is acute diffuse leukoencephalopathy, which can translate to give patient timely treatment and counseling.

## Author contributions

Data curation: Bing Liang.

Supervision: Chuanzhu Yan.

Visualization: Yi Zhou.

Writing–original draft: Yi Zhang.

Writing–review & editing: Cuiping Zhao.
